# Automatic assessment of adverse drug reaction reports with interactive visual exploration

**DOI:** 10.1038/s41598-022-10887-5

**Published:** 2022-04-26

**Authors:** Zongyang Gao, Yu Yang, Ruogu Meng, Jinyang Yu, Liang Zhou

**Affiliations:** 1 National Institute of Health Data Science, Peking University, 38 Xueyuan Road, Beijing, 100191 China; 2 Department of Mathematics, University of British Columbia, Vancouver, V6T1Z BC Canada; 3 Center for ADR Monitoring of Guangdong, Guangzhou, 510080 China

**Keywords:** Drug safety, Statistics

## Abstract

A large number of adverse drug reaction (ADR) reports are collected yearly through the spontaneous report system (SRS). However, experienced experts from ADR monitoring centers (ADR experts, hereafter) reviewed only a few reports based on current policies. Moreover, the causality assessment of ADR reports was conducted according to the official approach based on the WHO-UMC system, a knowledge- and labor-intensive task that highly relies on an individual’s expertise. Our objective is to devise a method to automatically assess ADR reports and support the efficient exploration of ADRs interactively. Our method could improve the capability to assess and explore a large volume of ADR reports and aid reporters in self-improvement. We proposed a workflow for assisting the assessment of ADR reports by combining an automatic assessment prediction model and a human-centered interactive visualization method. Our automatic causality assessment model (ACA model)—an ordinal logistic regression model—automatically assesses ADR reports under the current causality category. Based on the results of the ACA model, we designed a warning signal to indicate the degree of the anomaly of ADR reports. An interactive visualization technique was used for exploring and examining reports extended by automatic assessment of the ACA model and the warning signal. We applied our method to the SRS report dataset of the year 2019, collected in Guangdong province, China. Our method is evaluated by comparing automatic assessments by the ACA model to ADR reports labeled by ADR experts, i.e., the ground truth results from the multinomial logistic regression and the decision tree. The ACA model achieves an accuracy of 85.99%, a multiclass macro-averaged area under the curve (AUC) of 0.9572, while the multinomial logistics regression and decision tree yield 80.82%, 0.8603, and 85.39%, 0.9440, respectively, on the testing set. The new warning signal is able to assist ADR experts to quickly focus on reports of interest with our interactive visualzation tool. Reports of interest that are selected with high scores of the warning signal are analyzed in details by an ADR expert. The usefulness of the overall method is further evaluated through the interactive analysis of the data by ADR expert. Our ACA model achieves good performance and is superior to the multinomial logistics and the decision tree. The warning signal we designed allows efficient filtering of the full ADR reports down to much fewer reports showing anomalies. The usefulness of our interactive visualization is demonstrated by examples of unusual reports that are quickly identified. Our overall method could potentially improve the capability of analyzing ADR reports and reduce human labor and the chance of missing critical reports.

## Introduction

Drug safety has been well considered during the highly complex process of drug development, including phase I to phase III trials. However, pre-marketing trials lack sufficient power to reliably detect important adverse drug reactions (ADRs). Post-marketing surveillance for ADRs is indispensable since the possibility of rare, but serious ADRs or common delayed ADRs cannot be excluded even after approval to further ascertain safety for patient consumption. Post-marketing drug safety surveillance has primarily depended on spontaneous data from the ADR reporting system for decades, which relies on voluntary reporting by medical professionals, pharmaceutical companies, health authorities, or patients and their families^1^. The spontaneous reporting system (SRS) has been established in China since 1989 and supervision of ADRs now is a working priority for the drug authorities of China^2^.

The identification and evaluation of signals of possible ADRs have required careful manual review of individual case safety reports (ICSRs) from SRSs. Causality assessment is a fundamental feature of ICSRs review^3^. The WHO-UMC^4^ system for expert judgment is the most widely accepted method for causality assessment worldwide. The current approach for causality assessment of SRS in China was listed in the “Administrative Measures for the Reporting and Monitoring of Adverse Drug Reactions” revised by the Ministry of Health in 2011. This approach adapts the WHO-UMC system—it follows the basic principle of judging the causal relationship of adverse reactions and considers the timing, consistency, intensity, and specificity of adverse reactions. The correlation evaluation of adverse drug reaction (ADR)/adverse drug events (ADE) reports adopts the 6-level evaluation method with the following categories: “certain”, “probable/likely”, “possible”, “impossible”, “conditional/unclassified” and “unassessable/unclassifiable”.

Such assessment approaches highly depend on individual expertise and judgment, making it a knowledge- and labor-intensive task. China Food and Drug Administration (CFDA) had established ADR monitoring centers at different levels (municipal, provincial, and national levels) as technology supporting institutions^5^. However, not all reports would be reviewed by experts at different levels. According to the current regulation, only a small part of individual reports judged as serious ADR reports would be reviewed again by ADR experts from ADR monitoring centers at the provincial level every year. Moreover, only reports of death cases would be reviewed by national-level experts. For example, in 2020, 1676000 ADR reports were received in the SRS system, of which ADR experts reviewed only about 10%. The lacking of an effective tool for examining ADR data may have reduced the value of the large number of reports collected through the SRS.

In this paper, we advocate for assisting in reviewing and assessing ADR reports with automated assessment prediction and interactive visualization. Automatically assessing all reports could provide ADR experts with an overview of all reports and help ADR reporters to review and improve their reports. However, a perfect prediction model does not exist, and, therefore, automatic assessments cannot replace the judgment of humans. In fact, it is vital to have humans in the loop in drug safety practices. Interactive visualization is a human-centered technology that has demonstrated its usefulness and effectiveness in balancing the cognitive advantage of humans and the computational advantage of computers in data mining^6^ and decision making^7^. Therefore, we propose to use interactive visualization for users to explore ADR reports and examine reports of interest with their automatic assessments in detail. To facilitate filtering ADR reports with interactive visualization, a warning signal is devised such that characteristics of ADR reports could be conveniently measured quantitatively. Overall, our technique is a new approach for assessing and reviewing ADR reports in large quantities.

## Method

Unlike a large body of research focusing on “signal detection” from ADR reports, our work is intended to automatically assess the causality category of reports by learning from ADR experts and facilitate the exploration of ADR reports thereafter. In the remainder of this section, we will provide an overview of the method, and then, discuss each module of the method in detail, including the data, the automatic assessment model (ACA model), the warning signal computation, and the analysis with interactive visual exploration.

### Overview

**Figure 1:**
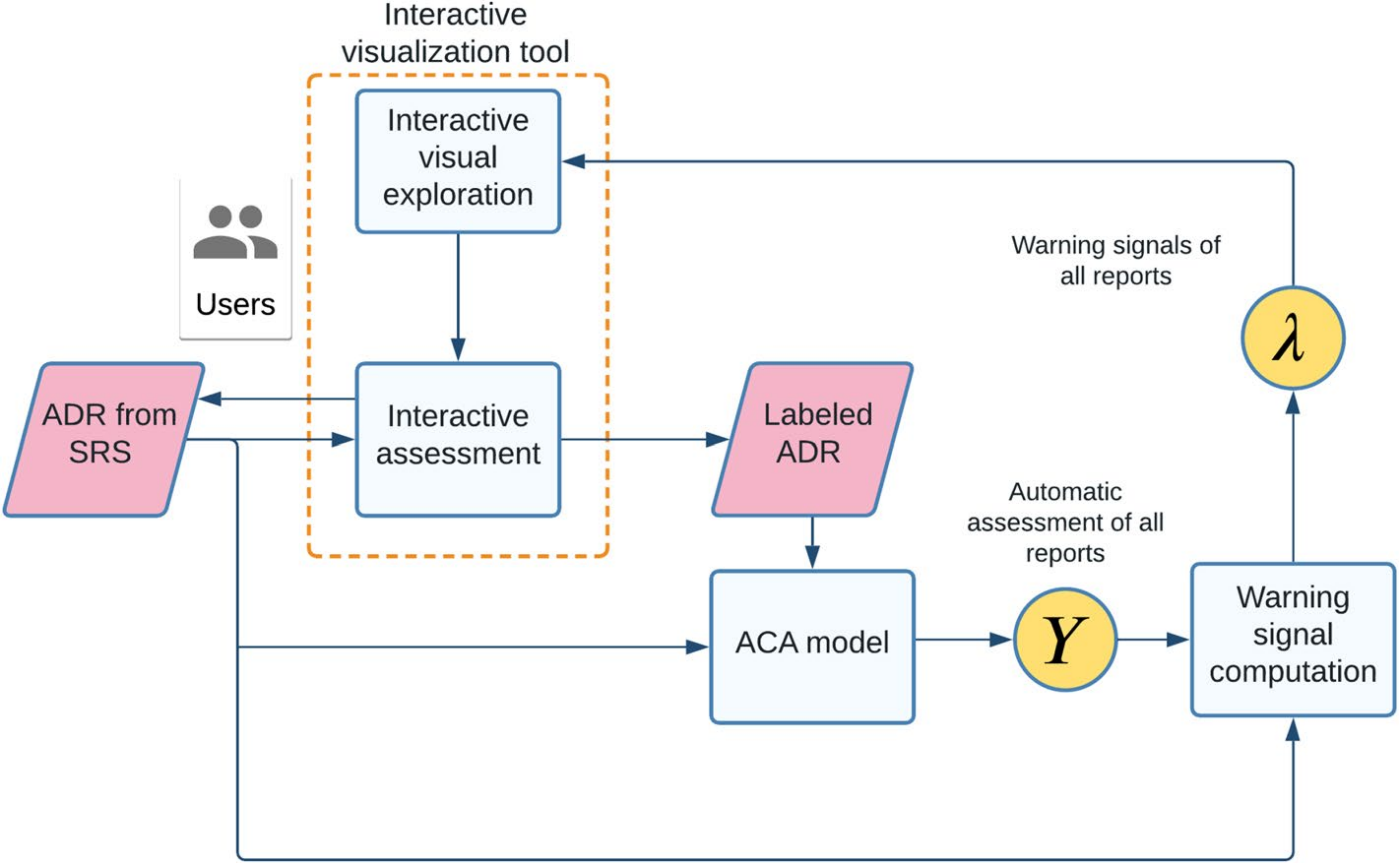
The workflow of our method.

The workflow of our method is shown in Fig. 1, which features an iterative human-in-the-loop approach. The input is report data from the SRS, which could be interactively assessed with the visualization tool (the orange box with dashed line) to provide labeled ADR reports. The labeled reports (typically, a small portion of all reports)—either assessed with or without the interactive visualization tool, e.g., previously manually assessed data—along with the input data are processed by our ACA model.

The ACA model predicts an assessment *Y* for each report in the SRS dataset. Our new warning signal  $\lambda$ is calculated for each record using *Y* and the original ADR data, and $\lambda$ is used for filtering so that users could focus on particular groups of reports, e.g., reports with high absolute values of the warning signal  as discussed in “Interactive visual exploration of the ACA model results”. The warning signal  $\lambda$ is used for interactive exploration of ADR reports in our visualization tool. Interactive exploration could improve the assessment and allows users to examine specific report more closely. Moreover, ADR reporters could easily compare the difference between model assessment and theirs and possibly improve the quality of assessments after that. Therefore, the workflow of our method forms a loop that iteratively improves the quality of SRS reports.

### Data

Spontaneous reports recorded by the Guangdong adverse drug reaction monitoring center in 2019 (2019 dataset, hereafter) that have been anonymized were used in our study under the approval by the institutional review board ethics committee of the Peking University with ethical exemption (IRB00001052-20012-exempt). The total number of records in the dataset amounts to 137964. Among all records, 20022 records labeled by ADR experts of Guangdong adverse drug reaction monitoring center were used for model training and testing. The data contain various types of variables: Boolean variables describing gender of the patient, the classification of ADR/ADE (new or not; serious or not); ordinal variables recording causality assessments of the report by the reporter, the institute of the reporter, experts of municipal ADR administration, and experts of ADR administration at provincial-level ADR experts; ADR/ADE occurrence time, the duration of the drug usage; textual information on the ADR/ADE symptoms, the brand name and the trade name of the drug, and other information required in formatted report.

The value of an assessment is categorized into five levels in an increasing order of certainty and coded as follows.

- 1 = unassessable/unclassifiable
- 2 = conditional/unclassified
- 3 = possible
- 4 = probable/likely
- 5 = certain

We use all variables in our interactive visualization tool and a subset of the variables are chosen for the automatic assessment model. Variables that are relevant for automatic assessment are first empirically selected by an ADR expert and then systematically refined by experimenting with the performance of the model. The resulting variables used for our automatic assessment model are listed and explained in Table 1.

**Table 1:** Variables used in the ACA model.

Name of variable	Description	Type
Casualty Assessment 1	Plausible time relationship to drug intake	Boolean
Casualty Assessment 2	Matched with known ADR types	Boolean
Casualty Assessment 3	Response to withdrawal plausible	Boolean
Casualty Assessment 4	Rechallenge satisfactory	Boolean
Casualty Assessment 5	Can be explained by other factors	Boolean
Severity Assessment 1	Death	Boolean
Severity Assessment 2	Carcinogenic, teratogenic, or birth defect	Boolean
Severity Assessment 3	Significant or permanent disability or damage to organ function	Boolean
Severity Assessment 4	Life threatening	Boolean
Severity Assessment 5	Admission or prolonged hospitalization	Boolean
Severity Assessment 6	Other significant medical events	Boolean

### Automatic causality assessment model

The prediction of assessments of the data could be realized by logistic regression. Multinomial logistic regression is a typical method for such a task^8^. However, this requires the fitting of a large number of parameters, so the degree of freedom used in the model fitting process will put excessive demands on the data set. More problematic, multiple logistic regression does not take into account the ordinal properties of the response variables^9^.

In contrast, ordinal logistic regression is geared for data with ordinal response variables. Moreover, ordinal logistic regression has no specific requirements on variable types, considering that all dependent variables are classified variables, it is a simple and applicable method in this situation. Often, ordinal logistic regression is achieved through the proportional-odds cumulative logit model^10^. The model reads:

1 $$\begin{aligned} {\text {logit}}[P(Y_i\le j)]&=\ln \frac{P\left( Y_i\le j\mid X_i\right) }{1-P\left( Y_i\le j\mid X_i\right) }=\theta _j + \beta ^{T}X_i,\nonumber \\ j&=1,2,3,4,5\quad i=1,\ldots ,n, \end{aligned}$$

where $Y_i$ is the ordinal dependent variable, $X_i$ is the independent variable, and *i* is the index of the record within the dataset of *n* records. The probability function of the proportional-odds model is:

2 $$\begin{aligned} P(Y_i\le j)=\frac{\exp (\theta _j+\beta ^{T}X_i )}{1-\exp (\theta _j+\beta ^{T}X_i )}, j=1,2,3,4,5\quad i=1,\ldots ,n, \end{aligned}$$

where $\theta _j$ is the cut point of the *j*th logit function. This model is based on the assumption that the slope coefficients do not depend on the selection of cut-off points^11^, i.e., the parallel assumption. However, the parallel assumption can be easily violated.

As a natural extension of the proportional odds model, the partial proportional odds model is proposed^10^. This model allows some variables to violate the parallel assumption, in other words, allows some to vary among logit equations. The partial proportional odds model can be expressed as follows:

3 $$\begin{aligned} {\text {logit}}[P(Y_i\le j)]&=\ln \frac{P(Y_i\le j \mid X_{i})}{1-P(Y_i\le j \mid X_i)}=\theta _j+\beta ^{T}X_i+\tau _j^{T}T_i, \nonumber \\ j&=1,2,3,4,5\quad i=1,\ldots ,n. \end{aligned}$$

The probability function reads:

4 $$\begin{aligned} P(Y_i\le j)&=\frac{\exp (\theta _j+\beta ^{T}X_i+\tau _{j}^{T}T_i)}{1- \exp (\theta _j + \beta ^{T}X_i+ \tau _{j}^{T}T_i)}, \nonumber \\ j&=1,2,3,4,5\quad i=1,\ldots ,n, \end{aligned}$$

where $T _ i$ is a $q\times 1$ vector, $q\le p$, represents the values of *q* independent variables violating the parallel assumption, and $\tau _ j$ is the regression coefficients associated with those variables, also depend on the selection of *j*. If all variables follow the parallel assumption, it becomes the proportional odds model.

Therefore, the partial proportional odds model is used in our ACA model as it is more flexible and has a higher tolerance of independent variables compared to the proportional odds model. In our model, $$ X _i$$ is the independent variable of *p* attributes, which is a $$p\times 1$$ vector, and $$Y_i$$ denotes the recorded causality category assessment of ordinal scales 1 through 5 of the *i*th report in the dataset that contains *n* records. Here, the number of *p* is determined to be 11 according to our variable selection as shown in Table 1.

We used the 2019 dataset that contains provincial-level ADR experts labeled records to train our model. The labeled data, i.e., the ground truth data, contains 20022 records in total, and we divide the records randomly into a training set (16,017 records) and a testing set (4005 records) to train the model. We implemented the ACA model in R aided by the ‘vgam’ package that readily implements the partial proportional odds model^12^. We compare the results of the ACA model to the ground truth data (expert labeled assessment), and those from the multinomial logistic regression and the decision tree methods. The accuracy, $F_1$ scores, and multiclass receiver operating characteristic (ROC) curves^13^ are calculated for each method. The multiclass ROC is used as the standard ROC is designed for binary classification, it is not suitable for our case of classification of multiple ordinal classes. The results can be found in “Results”.

### Warning signal computation

With our automatic assessment model, each report now has a predicted assessment trained with assessments by provincial-level ADR experts providing a reference for an ADR reviewer. However, exploring and analyzing the whole dataset even with automatic assessments is still challenging and laborious.

To this end, a warning signal that could quickly draw the attention of an ADR reviewer to reports of interest is helpful. Our goal is to encode the following information with the warning signal: I1, whether an agreement exists between assessments by the ACA model and a reporter, for example, both agree that a true adverse event occurred but assessments could vary on degrees; I2, the difference between the assessment of a reporter and the ACA model; I3, the severity of the adverse event. Moreover, the warning signal should use a medium value to encode the majority of reports, whereas high and low values indicate reports that are distinct as they are of potential interest.

We denote assessments from the ACA model and the spontaneous report as *Y*, and $Y^0$, respectively. The term *s* is designed to encode I1 and I3 using variables *d* and *r*:

5 $$\begin{aligned} d&= {\left\{ \begin{array}{ll} |Y - Y^0 |, (Y -3)(Y^0 - 3) > 0,\\ Y + Y^0 - 6, (Y -3)(Y^0 - 3) = 0,\\ -|Y - Y^0 |, (Y -3)(Y^0 - 3) < 0.\\ \end{array}\right. } \end{aligned}$$

6 $$\begin{aligned} r&= {\left\{ \begin{array}{ll} 1, {\text {normal ADE}},\\ 4, {\text {serious ADE}}, \end{array}\right. } \end{aligned}$$

where *d* describes the degree of disagreement of assessments (I1), and *r* describes the severity of the adverse event (I3). The variable *d* is designed in a way (Eq. 5) that a positive sign indicates that the assessment of ACA model agrees with that of the report, and a negative sign indicates that the two assessments disagree; whereas its absolute value measures the difference of the two assessments. To support intuitive user interactions in the visualization, *s* is designed to have a symmetric range by offsetting *d* by one ($d+1$) as in Eq. (8). The values of the term *r* are chosen to avoid the warning signal value overlapping for normal and serious ADE reports. As a result, a symmetric range is achieved for *s* with the possible values being a set $S = \{-16, -12, -8, -4, -3, -2, -1, 0, 1, 2, 3, 4, 8, 12, 16\}$. For a normal ADE, $s \in [-3,3]$, while $s \in [-16,-4] \cap [4,16]$ suggests a serious ADE.

At this point, I1 and I3 have been described with *s*, which has a symmetric range with severe cases spreading at two ends and normal cases at the center. To capture the difference information I2, we use another variable *t* that measures the difference between *Y* and $Y^0$. Therefore, our final warning signal  $\lambda$ is a tuple of *s* ($\lambda _s$) and *t* ($\lambda _t$) for each report:

7 $$\begin{aligned} \lambda&= (\lambda _s, \lambda _t) = (s, t), \end{aligned}$$

8 $$\begin{aligned} {\text {where}}\, s&= r\cdot (d+1), \end{aligned}$$

9 $$\begin{aligned} t&= Y - Y^0. \end{aligned}$$

With $\lambda$, a reviewer could efficiently and flexibly filter the reports, and focus on the few reports that are of interest. The usefulness of the warning signal is demonstrated with our interactive visualization in “Interactive visual exploration of the ACA model results”.

### Interactive visualization

Parallel coordinates^14,15^ are a widely-used multidimensional visualization technique that arranges data dimensions (variables) as parallel vertical axes and visualizes data as polylines across these parallel axes. We choose parallel coordinates as our interactive visualization method for its two advantages: data dimensions are readily scalable by simply adding more vertical axes, and multidimensional data query can be achieved by interactively selecting ranges, namely, brushing, on specific axes. The arrangement of axes in parallel coordinates could be also modified for visualizing correlation information of intended orderings of dimensions.

Our dataset of 137,964 reports imposes a challenge as they are too large for interactive visualization and manipulation if rendered as a whole at every update, i.e., every user selection, window resizing, or axis reordering. To achieve interactivity, we adopt progressive rendering that draws a portion of the full dataset at every frame and generates the full visualization progressively.

**Figure 2:**
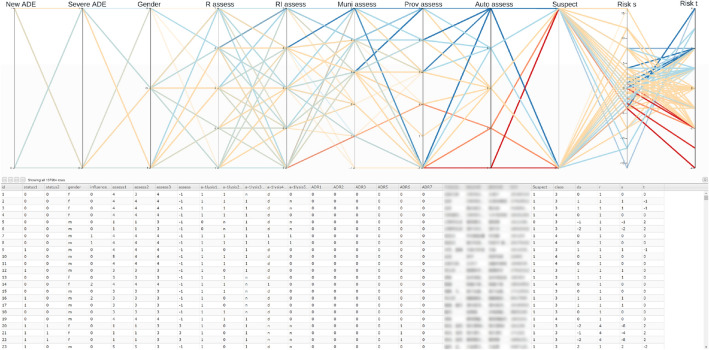
The interactive visualization tool with the ADR dataset of 137,964 records.

The user interface of our interactive visualization method is shown in Fig. 2. The parallel coordinates plot is shown on the top of Fig. 2 with ordinal and Boolean variables as axes with the following default ordering: known or unknown ADE (New ADE), normal or severe ADE (Severe ADE), Gender, the assessment of the reporter (R assess), the assessment of the reporter’s institute (RI assess), the assessment of the municipal-level regulator (Muni assess), the assessment of the provincial-level regulator (Prov assess), suspected or co-occurring drug (Suspect), automatic assessment (Auto assess), warning signal $\lambda _s$ (Risk s), and warning signal $\lambda _t$ (Risk t). Moreover, we use “RI assess” as the assessment $Y^0$. These configurations are empirically determined by an ADR expert. To facilitate the exploration, a linked table view (bottom of Fig. 2) is used for showing the full information of each ADR report including the textual information of symptoms, the product name, and the batch number. Polylines in the parallel coordinates are colored based on our predicted assessment *Y*—with a red-yellow-blue color map indicating the causality category 1 (red) through 5 (blue). The table view is linked with the parallel coordinates so that changes on one view is reflected on the other, e.g., updates of brushing on parallel coordinates change the visibility of corresponding data items in the table view, and focusing on one row of the table highlights the corresponding polyline in the parallel coordinates. Moreover, the table supports convenient editing of elements enabling users to assess ADR reports interactively.

Our visualization tool is an interactive web page that can be conveniently accessed by users with modern web browsers. The tool is implemented in HTML and JavaScript aided by the Data-Driven Documents (D3.js)^16^, d3-parcoords^17^, and the SlickGrid^18^ libraries.

### Ethical approval

All data used in this study are anonymous. All methods were performed in accordance with the relevant guidelines and regulations. This study have been approved by the institutional review board ethics committee of the Peking University and obtained ethical exemption (Ethical approval number: IRB00001052-20012-exempt).

## Results

Results of our method are shown through the performance analysis of the ACA model and reports of interest found through interactive visual exploration. The performance of the ACA model is evaluated by comparing it to the ground truth and other competing models in “The ACA model evaluation”, and the analysis of reports is discussed in “Interactive visual exploration of the ACA model results”.

### The ACA model evaluation

We compute the accuracy, the $F_1$-score, and multiclass ROC curves as performance measures for the evaluation. The ACA model is compared to the ground truth, the multinomial logistic regression (MLR), and the decision tree (DT) on the testing set (4005 records, i.e., 20% of the labeled data). Note that the same set of variables as in Table 1 are used in the multinomial logistic regression and the decision tree. Performance measurements of these methods are summarized in Table 2.

**Table 2:** Performance comparison of different assessment models on the test set.

Measurements	ACA model	MLR	DT
Accuracy	$85.99\%$	$80.82\%$	$85.39\%$
$F_1$-2 (conditional/unclassified)	0.4	0	0
$F_1$-3 (possible)	0.8329	0.7716	0.8346
$F_1$-4 (probable/likely)	0.8775	0.8482	0.8692
$F_1$-5 (certain)	0.9083	0	0.8559
AUC-macro	0.9572	0.8603	0.9440
AUC-micro	0.9748	0.9572	0.9779
AUC-2 (conditional/unclassified)	0.9878	0.9580	0.9416
AUC-3 (possible)	0.9324	0.9162	0.9398
AUC-4 (probable/likely)	0.9291	0.9126	0.9176
AUC-5 (certain)	0.9796	0.6547	0.9768

*ACA model* Automatic casualty assessment model, *MLR* Multinomial logistic regression, *DT* Decision tree, $F_1$-x: the $F_1$ score of category x (category 2–5), *AUC* Area under curve, *AUC-x* the multiclass AUC of category x (category 2–5).

The ACA model achieves an accuracy of $85.99\%$. In comparison, the multinomial logistic regression and decision tree achieve the accuracy as $80.82\%$ and $85.39\%$, respectively. The $F_1$-score that is widely used for performance measurement in classification problems is the harmonic mean of the precision and sensitivity. $F_1$-score can better measure the performance of models than the accuracy. In our case, $F_1$-score is calculated for each category. Note that category 1 was not used by provincial ADR experts leading to $F_1 = 0$ of category 1 for all methods, and are, therefore, omitted. As shown in Table 2, for category 2 (“conditional/unclassified”) through 5 (“certain”), the $F_1$ scores of our method are 0, 0.4, 0.8329, 0.8775, and 0.9083, respectively; multinomial logistic regression yields scores of 0, 0, 0.7716, 0.8482, and 0; scores of the decision tree are 0, 0, 0.8346, 0.8692, and 0.8559.

**Figure 3:**
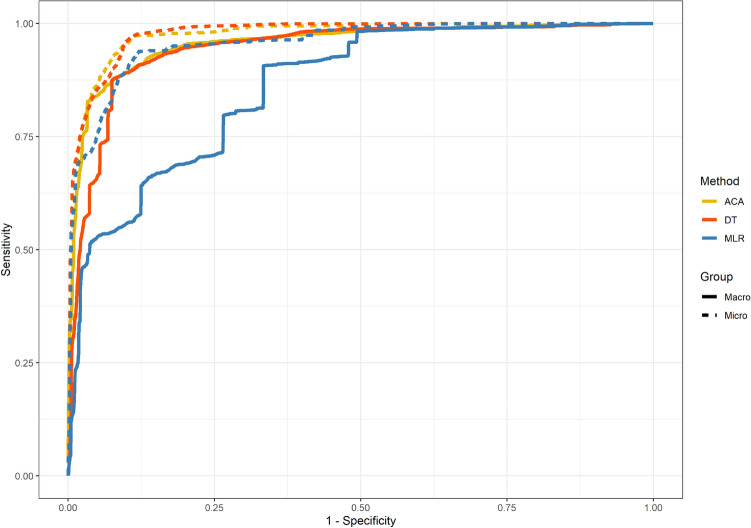
The macro and micro multiclass ROC curves of the ACA model (ACA), multinomial logistic regression (MLR), and decision tree (DT).

**Figure 4:**
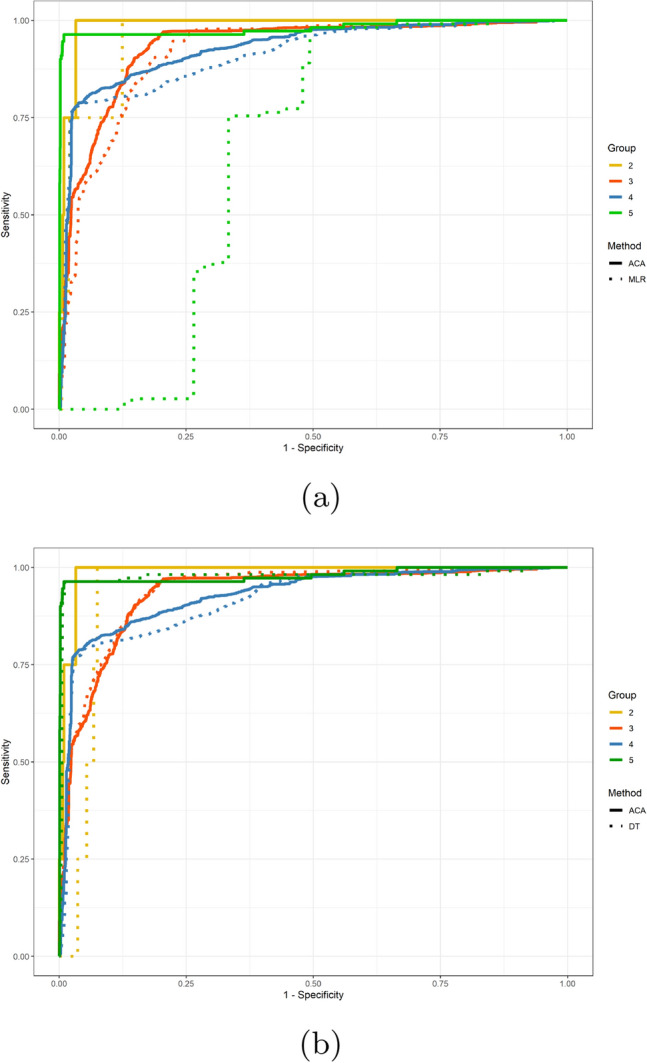
ROC curves of the ACA model (solid lines) and the multinomial logistic regression (**a** dash lines), and the decision tree (**b** dash lines) on causality categories.

Multiclass ROC curves and the associated AUC are calculated for further evaluation. The averaged ROC curves can be seen in Fig. 3, where micro curves—ROC curves calculated using true positives, false positives, true negatives, and false negatives of each class—are solid, and macro curves—the average of ROC curves of each class—are dashed^19^. The ACA model, the multinomial logistic regression, and the decision tree are drawn as blue, red, and yellow curves, respectively. Note that a baseline has to be set for multinomial logistic regression. Different baseline settings affect the performance of the multinomial logistic regression: when the baseline is set to 4 (“probable/likely”), the macro AUC is 0.8603, when the baseline is set to 5 (“certain”), the macro AUC is about 0.8, and the macro AUC is about 0.65 (the minimum is 0.5) when the baseline is set to categories 1, 2, 3 (“unassessable/unclassifiable”, “conditional/unclassified”, “possible”). Detailed comparison of each causality category of our method and the multinomial logistic regression is shown in Fig. 4a, while b shows the comparison of results of ours and the decision tree. The AUC of the macro and micro curves, and the AUC of each category for all methods are listed in Table 2.

### Interactive visual exploration of the ACA model results

Evaluations of the ACA model in the previous section show the performance of the new model numerically. In this section, reports of interest that are found with interactive visual exploration are examined by ADR experts to demonstrate the usefulness of the new warning signal $\lambda$. ADR experts are interested in reports that have high absolute values of $\lambda _s$ and $\lambda _t$ in combination with conditions of other variables. Such reports of interest are not easily detected with traditional methods in the dataset, but can be quickly and flexibly identified through interactive visual exploration with our automatic assessment and warning signal. According to the design of $\lambda _s$, we are interested in negative $\lambda _s$ with high absolute values as they indicate that the ACA model provides opposite assessment predictions than reporters.

**Figure 5:**
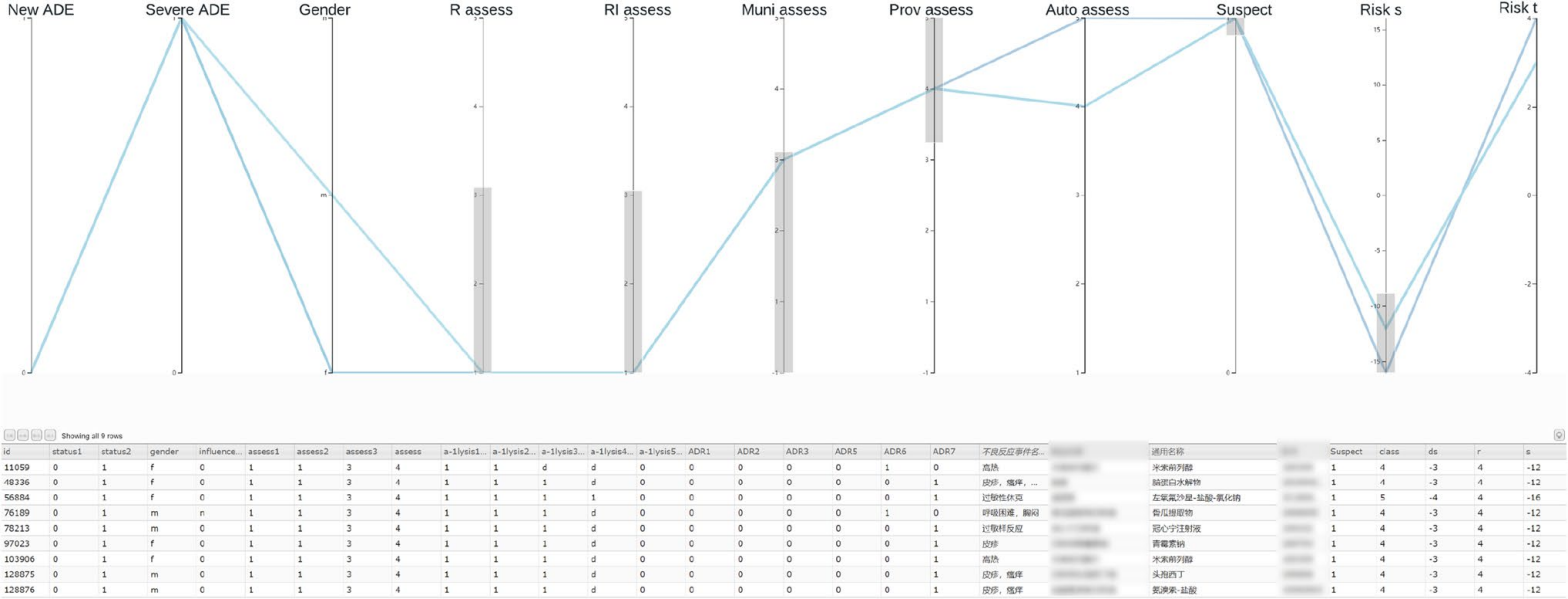
ADR reports with top (negative) scores of $\lambda _s$ (Risk s). These records are given opposite assessments by provincial experts and the ACA model as likely but possible or less possible by other assessments (causality category $\le 3$). The brushes on value ranges are shown as gray boxes on vertical axes.

By selecting suspect drugs (Suspect = 1) with negative $\lambda _s$ with high absolute values ($\lambda _s<-8$), 62 reports were found when we examined the whole records of 2019 dataset (137,964 reports). Among them, nine reports have opposite assessments between provincial-level experts (Prov assess) and reporters (R assess). The “Prov assess” is greater than 3, i.e., categories of probably/likely, or certain. In contrast, “R assess”, “RI assess”, and “Muni assess” are all less than or equal to 3, i.e., categories of unassessable/unclassifiable, conditional/unclassified, or possible. These reports can be easily found through interactive brushing on parallel coordinates as shown in Fig. 5, where brushes of range selections on variables are shown as gray boxes on vertical axes.

Details of the reports are summarized in Table 3. Notably, assessments by our ACA model (Auto) are almost identical to the Prov assess with one exception that a 5 is given by our method with the report of drug “Levofloxacin Hydrochloride”. All of the ADRs included had been reported previously (National medical products administration of China (https://www.nmpa.gov.cn/) and U.S. Food and drug administration (https://www.fda.gov/) were used for ADR information acquisition), and most of them were listed in the ADR Section of the instruction of drug.

**Table 3:** Reports of interest found through interactive visual exploration.

Adverse event	Drug name	*s*	Auto	R	RI	Muni	Prov
Fever	Misoprostol	$-12$	4	1	1	3	4
Rash, itching	Brain protein hydrolysate	$-12$	4	1	1	3	4
Anaphylactic shock	Levofloxacin hydrochloride	$-16$	5	1	1	3	4
Dyspnea, chest tightness	Bone melon extract	$-12$	4	1	1	3	4
Anaphylactic reaction	Guanxinning injections	$-12$	4	1	1	3	4
Rash	Penicillin sodium	$-12$	4	1	1	3	4
Fever	Misoprostol	$-12$	4	1	1	3	4
Rash, itching	Cefoxitin	$-12$	4	1	1	3	4
Rash, itching	Ambroxol hydrochloride	$-12$	4	1	1	3	4

*s*: warning signal $\lambda _s$, *Auto:* ACA model assess, *R:* R assess, *RI:* RI assess, *Muni:* Muni assess, *Prov:* Prov assess.

**Figure 6:**
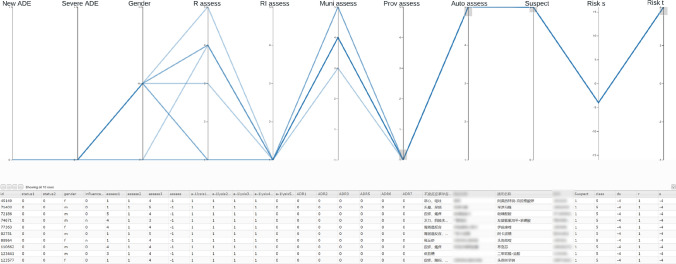
ADR reports with highest $\lambda _t$ (Risk t) and are not assessed by provincial ADR experts.

Reporters could also potentially benefit from using our method as the quality of their assessments may be improved even without assessments by higher-tier experts. Such a case is demonstrated in Fig. 6. We start by brushing on suspected drugs with no Prov assess (Prov assess = $-1$) in the 2019 dataset. Then, we select automatic assessment with the highest value (Auto assess = 5). As a result, ten records are found (see the table below the parallel coordinates in Fig. 5), and provincial-level ADR experts did not review those reports. Most of the event-drug pairs included in these ten reports were reported previously (with the aforementioned sources), but the assessment results between reporters, reporter’s institute, and the municipal-level regulator are inconsistent. Notably, assessments by reporter’s institute (RI assess) conflict with the municipal-level regulator: “RI assess” are “unassessable/unclassifiable” (RI assess = 1), whereas “Muni assess” vary from “possible” to “certain” (Muni assess $\ge 3$).

The analysis of reports with our warning signal  and the interactive visualization tool is efficient. In fact, the whole process of each example took less than one minute, so that the reviewer could quickly identify the reports of interest.

## Discussion

In this study, we successfully developed an automatic causality assessment model based on ordinal logistic regression. We combined this model with the interactive visualization tool to establish an iterative workflow that will help reporters for self-review and improvement. Our method is not a new model for ADR causality assessment but is to assist reporters at primary-level medical institutions and experts at municipal- and provincial-level alike in facilitating and improving the process of reporting and reviewing.

Logistic regression has been wildly used in medical research for years, where the typical logistic regression model is the binary logistic regression model. When possible confounding factors need to be controlled for, or multiple factors need to be considered, particular multivariate logistic regression could be used. However, typically used multinomial logistic regression requires the fitting of many parameters, so the degree of freedom used in the model fitting process will put excessive demands on the data set. Moreover, the multinomial logistic regression does not consider the ordinal properties of the output variables^9^. In contrast, ordinal logistic regression is devised for an ordinal response variable^8^. In addition, the ordinal logistic regression has no specific requirements on variable types, considering that all dependent variables are classified variables. Therefore, the ordinal logistic regression is an effective and applicable method for problems with ordinal outcomes. Ordinal logistic regression has been previously used in many areas including medical researches^20^, for example, in risk prediction and life expectation^21^. An ordinal risk index is fitted using ordinal logistic regression to predict the risk of postoperative acute kidney infection^22^. A risk score is modeled using ordinal logistic regression to predict hand-and-foot syndrome dynamics in a longitudinal study^23^. However, to our knowledge, ordinal logistic regression has not been used for causality assessment of ADR reports.

Visualization converts data into interactive graphics to gain insights into the data through perception and interaction. Medicine is an important research topic in visualization, and an overview of drug safety-related visualization methods can be found elsewhere^24^. A web-based tool with a visual interface is available for validating clinical cases and assessing detection rules of ADEs of hospital record data^25^. The tool relies on structured query language for setting ADE rules and generates reports with lab test results and drug prescriptions. A visual analytic method^26^ is proposed for analyzing ADR data with an overview visualization of ADEs, interactive filtering of co-occurring drugs, and an advanced query interface supporting hypotheses generation. This method supports signal generation based on the odds ratio using the contingency table. However, these methods do not analyze datasets with causality assessments nor support the assessment of such categories. A combined automatic and interactive visualization ADR assessment approach is not available currently.

Multinomial logistic regression and decision tree were chosen for comparison. Multinomial logistic regression model had been used to estimate the probability of severe ADRs^27^. And the first causality assessment method for drug-induced liver injury was based on the decision tree^28^. As shown in “The ACA model evaluation”, our ACA model achieved similar accuracy with the decision tree method and is better than multinomial logistic regression. The results of $F_1$ scores for categories 2–5 (“conditional/unclassified”, “possible”, “probable/likely”, “certain”) of our method are superior to the multinomial logistic regression for all categories and $F_1$ scores of categories 2 (“conditional/unclassified”) and 5 (“certain”) for the multinomial logistic regression are 0; our method has comparable $F_1$ scores of categories 3–5 (“possible”, “probable/likely”, “certain”) to the decision tree, however, a 0 $F_1$ score is achieved by decision tree for category 2 (“conditional/unclassified”).

Multiclass ROC curves are commonly used as an evaluation method for multiclass regression models, it can reveal True Positive Rate (TPR) and False Positive Rate (FPR) for each paired classes^19^. Results of multiclass ROCs show that the ACA model has better AUC than multinomial logistic regression for all curves (micro, macro, categories 1–5). Furthermore, the ACA model has comparable or slightly better AUC than decision tree for the micro, macro, category 3–5 (“possible”, “probable/likely”, “certain”) curves, and better AUC for categories 1 (“unassessable/unclassifiable”) and 2 (“conditional/unclassified”).

There are still some limitations of our method. First, only reports from one province of China in one calendar year were used to train and test the ACA model. The generality of the ACA model requires further studies. We have performed a preliminary study of the ACA model in spontaneous reports collected by the Guangdong adverse drug reaction monitoring center in 2018 (anonymous data with 143,406 reports under the same ethical approval as of the 2019 data) and received a comparable accuracy of $85.77\%$. Second, unbalanced data, significantly, when many categories exist, could impact the performance of ordinal logistic regression. The predicted results are potentially biased due to considerable differences between sample sizes of categories^29^, which is the nature of ADR reports. Third, the variables used in the ACA model were selected based on the experience of ADR experts. An automatic selection method should be used to choose variables systematically in the future. Last, the current visualization method is effective but does not cover the many aspects of the ADR data, and, therefore, we plan to design a full-fledged integrated visual analysis method in the future.

## Conclusion

In this paper, an automatic assessment method with interactive visualization exploration is proposed to aid the causality assessment of ADR reports. The ACA model automatically predicts causality assessment for spontaneous reports by learning assessments of experienced ADR experts. A warning signal  is derived based on the automatic assessments for filtering a large volume of reports. A parallel coordinates-based visualization method is used for interactive exploration of the ADR data extended by the automatic assessment and the warning signal. The ACA model achieves good accuracy, and potential reports of interest can be quickly and conveniently selected with our web-based interactive visualization tool. Our overall method could improve the efficiency of the assessment process and increase the coverage of ADR reports reviewing for ADR experts. Moreover, the method could also potentially improve the capability of analyzing ADR reports and is promising in reducing human labor and the chance of missing critical reports.

For future work, we would like to further improve the model for better prediction efficiency and automate the variable selection process. Another direction is to devise a comprehensive interactive visual analysis method to provide users an integrated environment for more efficient review and assessment of ADR reports and to gain more insights during the process.
